# Supplementary material for: Type 1 dopamine receptor (D1R)-independent circadian food anticipatory activity in mice
Source: PLoS One. 2021 Feb 8;16(2):e0242897. doi: 10.1371/journal.pone.0242897 (PMC7869994; doi:10.1371/journal.pone.0242897)
Supplement: Attachment — Submitted filename: D1R revision cover letter_.docx [file pone.0242897.s002.docx]

Andrew Steele, PhD

Associate Professor

Biological Sciences Department

California State Polytechnic University, Pomona

adsteele@cpp.edu

(909) 869 2480

December 30, 2020

Dear Dr. Bartell,

Thank you for serving as the editor for our manuscript (PONE-D-20-35295, Type 1 dopamine receptor (D1R)-independent circadian food anticipatory activity in mice). Below please find our responses to your assessment and to the specific points brought up by the reviewers. We wrote our responses in **bold font** to make them more apparent.

Please do not hesitate to contact me with questions or concerns.

Sincerely,

Andrew D. Steele, PhD

Thank you for submitting your manuscript to PLOS ONE. After careful consideration, we feel that it has merit but does not fully meet PLOS ONE’s publication criteria as it currently stands. Therefore, we invite you to submit a revised version of the manuscript that addresses the points raised during the review process.

**Thank you--we appreciate the opportunity to submit a revised manuscript.**

It is important that, in light of the sex differences that have been observed in FAA, that the authors analyze their data for differences among sexes.

**We agree that it is important to examine sex differences and present a full response to this issue below.**

2. To comply with PLOS ONE submissions requirements, please provide methods of sacrifice in the Methods section of your manuscript.

**We have added the detail that the mice were euthanized with CO_2_ gas.**

3. Please include your tables as part of your main manuscript and remove the individual files. Please note that supplementary tables should be uploaded as separate "supporting information" files.

**We have included the tables as part of the main manuscript file.**

4. Thank you for stating the following in the Acknowledgments Section of your manuscript:

"Funding was provided by the Whitehall Foundation, California State

Polytechnic University Pomona, CSU Program for Education and Research in

Biotechnology, and the National Institute of General Medical Sciences of the

National Institutes of General Medical Sciences of the National Institutes of Health

under Award Number SC3GM125570."

We note that you have provided funding information that is not currently declared in your Funding Statement. However, funding information should not appear in the Acknowledgments section or other areas of your manuscript. We will only publish funding information present in the Funding Statement section of the online submission form.

Please remove any funding-related text from the manuscript and let us know how you would like to update your Funding Statement. Currently, your Funding Statement reads as follows:

"National Institute of General Medical Sciences of the National Institutes of General Medical Sciences of the National Institutes of Health under Award Number SC3GM125570 to AS. The content is solely the responsibility of the authors and does not necessarily represent the official views of the National Institutes of Health."

Please include your amended statements within your cover letter; we will change the online submission form on your behalf.

**My apologies: I have removed the funding information from the Acknowledgments section. Please add the following line to the funding section: “The Whitehall Foundation provided a Research Grant to AS.”**

Reviewers' comments:

Reviewer's Responses to Questions

Comments to the Author

1. Is the manuscript technically sound, and do the data support the conclusions?

The manuscript must describe a technically sound piece of scientific research with data that supports the conclusions. Experiments must have been conducted rigorously, with appropriate controls, replication, and sample sizes. The conclusions must be drawn appropriately based on the data presented.

Reviewer #1: Partly

Reviewer #2: Yes

2. Has the statistical analysis been performed appropriately and rigorously?

Reviewer #1: No

Reviewer #2: Yes

**We are unsure of how to perform a more appropriate or rigorous statistical analysis. We consulted with Dr. Jeremy Claisse, who is a biostatistician in our department and he recommended the tests that we used (mixed-effects model). A two-way ANOVA yielded almost identical results in terms of statistical significance, but was not appropriate because of missing values at some time points.**

3. Have the authors made all data underlying the findings in their manuscript fully available?

**We have uploaded supporting information (Excel file titled “raw data organized by figure”) that contains all the data presented in the Figures.**

Reviewer #1: Yes

Reviewer #2: Yes

4. Is the manuscript presented in an intelligible fashion and written in standard English?

PLOS ONE does not copyedit accepted manuscripts, so the language in submitted articles must be clear, correct, and unambiguous. Any typographical or grammatical errors should be corrected at revision, so please note any specific errors here.

Reviewer #1: Yes

Reviewer #2: Yes

5. Review Comments to the Author

Please use the space provided to explain your answers to the questions above. You may also include additional comments for the author, including concerns about dual publication, research ethics, or publication ethics.

Reviewer #1: The authors used several gene ablation approaches to study the role of D1 dopamine receptor signaling in food anticipatory activity in mice fed 60% of their average ad lib food intake. In all, their data reveal a small effect of loss of D1DR signaling on the level of activity prior to daily feeding. Although not addressed in the paper, these manipulations appeared not to influence the timing of increased activity. This is nicely written paper attempting to shed new light on a long-standing issue in the study of circadian entrainment by feeding schedules, specifically the mechanisms that mediate the heightened activity observed prior to feeding. Unfortunately, in its present form it falls short of adding significant information on the topic. In particular, all experiments were carried-out in both male and female mice (as they should!). However, the reported results do not distinguish between the sexes and data from males and females are pulled together. This is an important issue given evidence of sex differences in food anticipatory activity, and evidence of an important role of female sex hormones on striatal DA receptor-mediated signaling. To gain better insight into the importance of D1DR signaling in FAA, and possibly to discover sexual dimorphism in their contribution, the authors are encouraged to reanalyze and report the data for males and females separately.

**We agree that it is important to examine sex differences (and to study both male and female biology). At the midway point of our study, we did an interim analysis to examine sex differences. At that point, there was not a trend toward obvious sex differences relatingi to deletion D1R and it seemed that sex differences would not affect the conclusions of our study--namely, that various D1R knockouts have an impairment in FAA but that the impairment is more subtle than we reported previously. It did not seem that the female D1R KO mice were worse off than the males or vice versa. This is in contrast to what our colleague, Ralph Mistlberger observed  when examining FAA using running wheels, where female D1R KO mice clearly had lower FAA than D1R KO males (Michalik et al., 2015; *Behavioral Neuroscience* 129 (3): 351–360).**

**In our laboratory’s prior investigation of sex differences and FAA, using home-cage behavior recognition software, we observed no sex differences in terms of total pre-meal activity if we asked “do males or females have higher levels of premeal activity in terms of seconds of behavior?” (Aguayo A, et al. 2018; *PLoS ONE* 13(1): e0191373) It was only upon normalization of the data that the sex difference became apparent; the normalization asks “of the activity that males/females had, how much of it proportionally was premeal?” Since females had a higher nighttime activity peak, their normalized FAA was lowered by the normalization. I am mentioning this because the sex differnce is nuanced and although our laboratory places the most weight on normalized FAA data, others in the field are less concerned with normalization and prefer to use absolute activity.**

**All that being said, to show that we took the suggestion of the reviewer seriously and to double-check for ourselves, we present below the normalized FAA data examining the 4 D1R KO strains separated by sex. Given that the sex-separated data has low sample sizes in some cases (i.e. is underpowered) and that the trends for sex differences are very weak we implore you to allow us to present the pooled male + female data in the main text of the manuscript as originally submitted. If the reviewers deem it appropriate, we could include the single sex data as a supplemental figure, but would prefer not to given that differences in samples sizes are just as likely to contribute to the effects (or lackthereof) that we have presented below.**

**We are no longer breeding any of these lines, so it would not be possible for us to expand the study and add additional males and females. We want to be clear that we agree with the reviewers/editor that in an ideal situation we would examine a large cohort (n>10 per group of female D1R KO, male D1R KO etc) but in this case we did not have the resources and it was also clear from an interim analysis that we conducted that it would not have modified the major conclusions of our study: that D1R KO mice have an impairment in FAA but that it is smaller than previously estimated.**

Reviewer #2: This is a follow up study on Gallardo et al. 2014 published by the same group. In the initial study, they found marked reduction in FAA upon global DRD1 KO. Here, the authors now conclude that the reduction is rather moderate suggesting that other dopamine receptors might contribute to FAA. In support of their revised claim, the authors present FAA data on Vgat-Cre FloxD1R, global D1RKO, global D1RKO resurrected from cryopreserved embryos, and a different D1RKO line.

The authors used timed 60% caloric restriction to induce FAA assessed by quantifying video-taped ‘high’ activities (jumping hanging rearing ambulating) during the 2hrs prior food replenishment which occurred at ZT6.

The authors found statistical differences in the absolute and relative amounts of FAA in all 4 models, however the dot plots clearly indicate that a fraction of the KO animals still showed FAA more or less indifferent from controls which is also acknowledged by the authors.

The authors confirm that D1 receptor expression is undetectable in the striatum across all models used, arguing against inefficiencies in gene disruption to explain the ‘milder’ phenotype.

Based on these findings, the authors arrive at the conclusion that DRD1 signalling contributes to FAA manifestation but that other dopamine receptors are likely involved as well.

This work is important as it revises previous findings central to the topic of food anticipation, even though the revision is slight.

Perhaps more importantly, the presented data puts the notion of an involvement of DRD1 in FAA on a stronger footing due to the use of different models reporting largely similar deficits.

**We appreciate and agree with the reviewer’s comment that although our results were initially disappointing, it does put D1R signaling on a more secure footing given that all 4 knockout strains showed an impairment in FAA.**

Comments:

As the Cryo D1KO showed the strongest phenotype it can be argued that some genetic drift perhaps happened, the authors may want to discuss this point (it seems a bit buried). The other genetic model, is it a transcriptional stop knock in? Some detail would be helpful here, considering that stops can be leaky (although the immuno indeed tells otherwise, at least as far as detectable DRD1 levels go).

**This is a good point. To do a more rigorous analysis of the % impairment in 4 KO lines, we pooled the control data into one large control group and plotted the normalized FAA data for the mutants. Surprisingly, the D1R KO^Drago-cryo^ line did not appear any more impaired than the D1R KO^Drago^ line; in fact, at day 21 of CR the D1R KO^Drago-cryo^ line was not significantly different from the pooled controls) whereas the D1R KO^Drago^ was significantly decreased at days 14, 21, and 28. Thus, we could not come out too strongly in favor of drift. We do not plan to include the data below in the revised manuscript but can do so if it is requested by the reviewer.**

Are there any GABA- DRD1 neurons? This should be perhaps addressed in the discussion.

**Given how complete the vgat-Cre deletion of D1R appeared, we do not suspect a GABA- D1R+ population (as this would have left D1R staining intact in those cells). I spent some time going through the publications describing the creation of the dopamine receptor reporter lines (D1-tdTomato and D2-eGFP) and Cre lines for D1 and D2 crossed to reporter lines (Gong et al., 2003; Valjent et al., 2009; Wang et al., 2015; Cheng et al., 2016; Gong et al., 2007; Kravitz et al., 2012; Pascoli et al., 2012), and did not find any evidence of GABA- D1R+ populations.**

I am not quite sure if the lack of obtaining KO offspring from the D1-Cre floxd1 cross should be mentioned in the abstract. The conclusion that the Cre transgene landed on the D1 encoding chromosome seems most parsimonious given that the D1KO phenotypes described in the literature are quite distant from embryonic lethality. Thus being likely only a technical issue, a brief mentioning in the results should suffice.

**We agree and have removed the sentence from the abstract mentioning the embryonic lethality of D1-Cre; D1R flox/flox mice.**

“The location of the neurons responsible for the anticipation of food and other related stimuli have been elusive”

Do we know if FAA relies on neurons solely? The FAA generator/mediator might include other cells and tissues….

**This is a good point and, in fact, it appears that the liver might be a major site of signaling for FAA--we cited a paper by Chavan and colleagues (2016; *Nature Communications* 10580) demonstrating that liver-derived ketones appear to be necessary for expression of FAA. That being said, at some level the brain must be involved to get the animals up and moving prior to mealtime. It is our goal to identify the neurons (and possibly glia) required for FAA but certainly contributions from other tissues are important, too.**
